# Efficient deep-blue LEDs based on colloidal CsPbBr_3_ nanoplatelets meeting the Rec.2020 standard

**DOI:** 10.1038/s41377-025-02019-1

**Published:** 2025-09-22

**Authors:** Yusheng Song, Sheng Cao, Yijie Wang, Mingyan Chen, Yu Zhang, Qiuyan Li, Shulin Han, Yi Liang, Lei Cai, Jialong Zhao, Bingsuo Zou

**Affiliations:** 1 School of Physical Science and Technology, State Key Laboratory of Featured Metal Materials and Life-cycle Safety for Composite Structures, Guangxi University, Nanning, 530004 China; 2 Hubei Key Laboratory of Energy Storage and Power Battery, School of Optoelectronic Engineering, School of New Energy, Hubei University of Automotive Technology, Shiyan, 442002 China; 3 Shandong Province Key Laboratory of Medical Physics and Image Processing Technology, School of Physics and Electronics, Institute of Materials and Clean Energy, Shandong Normal University, Jinan, 250014 China; 4 School of Resources, Environment and Materials, Guangxi University, Nanning, 530004 China

**Keywords:** Inorganic LEDs, Photonic devices

## Abstract

Colloidally quantum-confined CsPbBr_3_ nanoplatelets (NPLs) exhibit narrow emission linewidths and thickness-tunable photoluminescence, making them ideal candidates for deep-blue perovskite light-emitting diodes (PeLEDs). However, the weak surface coordination of conventional long-chain ligands (*e.g*., oleylamine and oleic acid) leads to face-to-face stacking of the NPLs, resulting in undesirable emission redshifts in their PeLEDs. Herein, we report an efficient deep-blue PeLED based on colloidal CsPbBr_3_ NPLs that meet the Rec.2020 color standard, enabled by an acid-assisted ligand passivation strategy. Surface chemical analysis reveals that hydrobromic acid facilitates proton-assisted stripping of the long-chain ligands, followed by the formation of stable Pb-S-P coordination bonds with thio-tributylphosphine, which exhibits a high adsorption energy (E_ads_ = -1.13 eV). This approach significantly improves surface defect passivation, yielding a photoluminescence quantum yield of 96% and a narrow 13 nm full-width-at-half-maximum deep-blue emission. Enhanced exciton recombination and reduced defect state density are evidenced by a prolonged photoluminescence lifetime and slower absorption bleach recovery kinetics. The resulting PeLEDs achieve record-breaking performance among CsPbBr_3_ NPL-based systems, with a maximum external quantum efficiency of 6.81% at 461 nm, a peak luminance of 143 cd m^-2^, and the CIE color coordinates (CIE-y = 0.046) that comply with Rec.2020 standards. This work presents an effective strategy for developing efficient and stable deep-blue perovskite emitters, demonstrating significant potential for the commercialization of perovskite nanomaterials in next-generation ultra-high-definition displays.

## Abstract (web summary)

Acid-assisted short-chain thio-tributylphosphine ligand passivation improves the surface stability of colloidal CsPbBr_3_ nanoplatelets, enabling deep-blue PeLEDs with CIE coordinates (0.136, 0.046) that meet the Rec.2020 standard.

## Graphical abstract

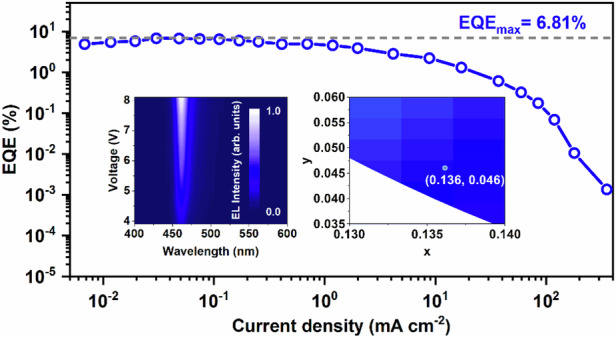

## Introduction

Metal halide perovskite light-emitting diodes (PeLEDs) have emerged as promising candidates for next-generation high-definition displays due to their wide color gamut and narrow full-width-at-half-maximum (FWHM)^1–3^. While recent research has primarily focused on enhancing device efficiency, achieving external quantum efficiencies (EQEs) over 20% for red^4^ and green^5^ PeLEDs and exceeding 15% for sky-blue variants^6^, less attention has been given to the precise control of color coordinates as defined by the Commission International de l'Éclairage (CIE). However, for ultra-high-definition applications, high efficiency alone is insufficient; accurate color tuning, especially of the three primary colors, is essential. This is particularly important for blue PeLEDs, which must meet the CIE-y ≤ 0.046 threshold specified by the International Telecommunication Union Recommendation Rec.2020 standard^7^. Despite significant advancements, achieving both high efficiency and spectrally deep-blue emission in PeLEDs remains a considerable challenge.

A direct approach to shifting the emission of PeLEDs toward the deep-blue region involves halide anion mixing (Br^-^/Cl^-^), which effectively widens the emitter material’s bandgap, shifting it from green to blue^8,9^. However, mixed-halide perovskites are intrinsically unstable due to the low formation energy of Cl^-^ vacancies^10,11^. This instability results in a high density of halide defects and enhanced ionic migration. Under operational electric fields, these instabilities lead to severe phase segregation, causing a redshift in PeLED emission, degrading color purity, and reducing operational stability^12–14^. As an alternative, Ruddlesden-Popper (RP) phase perovskites achieve blue emission through dielectric ligand-induced bandgap modulation^15–17^. Nevertheless, strong van der Waals interactions between bulky organic cations tend to stabilize low-n phases (e.g., *n* = 1), while short-chain ligands promote high-n phases, resulting in significant phase heterogeneity^18^. The coexistence of perovskite domains with varying bandgaps fosters undesired charge transfer, broadening the PeLED emission spectra and ultimately compromising spectral purity^19,20^.

Recently, quantum confinement in colloidal perovskite nanocrystals has emerged as a promising strategy for achieving narrowband deep-blue emission in PeLEDs^21–24^. Specifically, three-monolayer-thick CsPbBr_3_ nanoplatelets (NPLs) exhibit strong quantum confinement, emitting in the 460-470 nm range with narrow FWHM^25,26^, making them well-suited to meet color gamut standard requirements. However, conventional long-chain ligands such as oleic acid (OA) and oleylamine (OAm) provide weak surface binding, leading to poor colloidal stability^27,28^. During solution processing, ligand desorption promotes NPL fusion, reducing quantum confinement and causing redshifted emission above 480 nm (CIE-y > 0.1), thereby degrading color purity in PeLED applications^29,30^. Moreover, the high surface-to-volume ratio of NPLs exacerbates surface defect formation, increasing nonradiative recombination and reducing luminescent performance^31,32^. While cationic polymer capping has been explored to enhance film stability, it often impedes charge transport, ultimately limiting PeLED efficiency^33^. In contrast, recent advances in quantum dot systems have shown that hydrohalic acid-assisted surface passivation can effectively suppress surface defects and improve film ordering, leading to enhanced PeLED performance^34,35^. Inspired by these findings, a rationally designed acid-assisted ligand passivation strategy could offer a promising approach to simultaneously improve both the optical performance and operational stability of CsPbBr_3_ NPLs. Such a strategy holds the potential to enable the realization of high-performance deep-blue PeLEDs that meet the stringent Rec.2020 color gamut requirements, an area that remains largely unexplored.

In the present work, we report a hydrobromic acid-assisted ligand passivation strategy to synthesize high-quality CsPbBr_3_ NPLs for deep-blue-emitting PeLEDs. The passivated NPLs exhibit narrowband emission centered at 461 nm with a FWHM of just 13 nm and a remarkable enhancement in photoluminescence quantum yield (PL QY) from 19% to 96%. These NPLs also demonstrate excellent spectral stability (CIE-y ≤ 0.046) maintained over 60 days. In contrast, unpassivated control samples show additional emission peaks arising from face-to-face stacking due to ligand loss. Time-resolved PL and transient absorption spectroscopy reveal that the passivation suppresses nonradiative recombination pathways, extending the average exciton lifetime from 4.79 ns to 5.84 ns. This improvement is attributed to the formation of stable Pb-S-P surface bonds that mitigate bromide vacancy-related defects and incomplete octahedral coordination. As a result, the fabricated PeLEDs demonstrate state-of-the-art performance among CsPbBr_3_ NPL-based devices, achieving a peak EQE of 6.81%, a luminance of 143 cd m^-^^2^, and a color coordinate (0.136, 0.046), fully satisfying the Rec.2020 standard and representing a tenfold improvement over unpassivated control devices.

## Results

### Synthesis and optical properties of CsPbBr_3_ NPLs

**Fig. 1:**
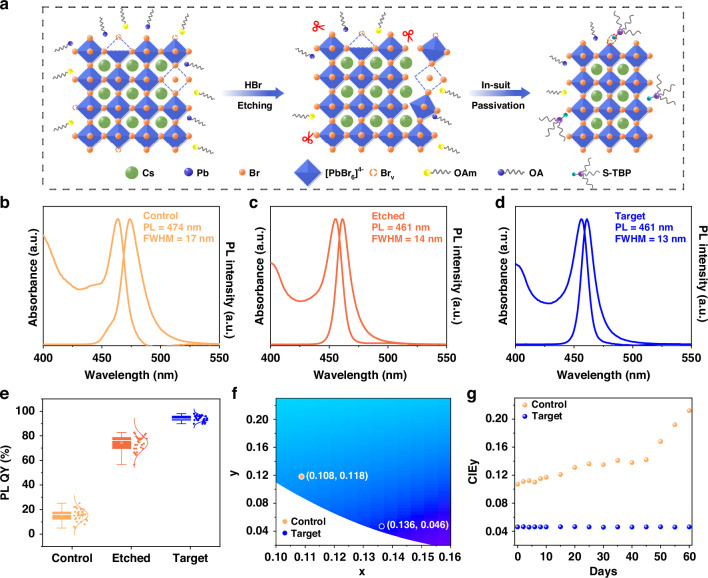
Optical properties of CsPbBr_3_ NPLs. **a** Schematic illustration of the HBr-assisted removal of loosely bound long-chain ligands and incomplete octahedral structures, followed by efficient surface vacancy passivation using novel short-chain S-TBP ligands. UV-vis absorption and PL spectra of **b** control, **c** etched, and **d** target NPLs. **e** Statistical distribution of PL QYs measured across 30 independent synthetic batches for control, etched, and target NPLs. **f** CIE 1931 chromaticity coordinates of control and target CsPbBr_3_ NPL colloidal solutions. **g** Long-term stability of the CIE-y coordinate for control and target NPL colloidal solutions under ambient conditions

Blue CsPbBr_3_ NPLs were synthesized using a modified thermal injection method with OA and OAm as capping ligands (details are provided in the experimental section). To address the weak dynamic binding properties of these ligands, which led to directional fusion-induced emission redshifts in PeLEDs, we employed a hydrobromic acid (HBr)-assisted ligand passivation strategy to enhance the spectral stability of CsPbBr_3_ NPLs. As shown in Fig. 1a, we introduced an appropriate amount of HBr during the nucleation stage of the NPLs. First, the protons from HBr effectively dissociated the long-chain ligands^36^ (Supplementary Note S1, Supplementary Information) and eliminated the incomplete octahedral structure on the surface while preserving the quantum confinement effect. Second, the Br^-^ ions from HBr precisely filled the halogen vacancy defects on the surface of the CsPbBr_3_ NPLs. To further optimize the surface passivation, we introduced a thio-tributylphosphine (S-TBP) ligand, which binds to the active surface sites exposed after dissociation of the long-chain ligands, facilitating a more efficient ligand exchange process.

To evaluate the impact of the acid-assisted ligand passivation strategy on the optical properties of CsPbBr_3_ NPLs, we characterized several samples using UV-visible absorption and PL spectroscopy. As shown in Fig. 1b, the untreated control sample (control CsPbBr_3_ NPLs) exhibits the strongest exciton emission peak at 474 nm, with a FWHM of 17 nm. After HBr etching, the CsPbBr_3_ NPLs experience a 13 nm blue shift, with the exciton emission peak shifting to 461 nm and the FWHM narrowing to 14 nm (Fig. 1c). This blue shift can be attributed to the introduction of HBr, which inhibits the Ostwald ripening process, thereby reducing the NPL size. Notably, the S-TBP passivated sample (target CsPbBr_3_ NPLs) shows the sharpest exciton emission peak at 461 nm, with an even narrower FWHM of 13 nm (Fig. 1d). PL QY measurements across 30 independent synthetic batches show significant improvement, increasing from 19% for the control NPLs to 78% after etching, and reaching 96% after S-TBP ligand treatment (Fig. 1e). In Fig. 1f, the target NPLs exhibit a CIE-y value of 0.046, substantially lower than the control NPLs (0.118). The color purity calculation shows that the color purity of the target NPLs increases from 87% to 99% (Supplementary Note S2, Supplementary Information), reflecting a significant improvement and meeting the stringent Rec.2020 standard for deep blue CIE color coordinates. These results demonstrate that the acid-assisted ligand passivation strategy effectively reduces surface defects while preserving quantum confinement in CsPbBr_3_ NPLs, resulting in enhanced optical properties.

To evaluate the improvement in stability of CsPbBr_3_ NPLs achieved through the acid-assisted ligand passivation strategy, we monitored the stability of both NPL solution and film under ambient environmental conditions (25 ± 5 °C, 55 ± 20% RH) for 60 days. The results showed that the PL QY of the control NPL solution decreased to less than 50% of its initial value within 10 days (Supplementary Fig. S1, Supplementary Information), accompanied by a visible color change from blue to green under UV irradiation (Supplementary Fig. S2, Supplementary Information). This degradation was further evidenced by the emergence of additional PL peaks (Supplementary Fig. S3, Supplementary Information) and an increase in the CIE-y coordinate from 0.118 to 0.212 (Fig. 1g). These changes are likely attributed to the detachment of surface ligands and directional fusion of the NPLs caused by unstable surface states^37^. In contrast, the target NPL solution maintained a high PL QY of 71% even after 60 days (Supplementary Fig. S1, Supplementary Information), along with stable PL spectra (Supplementary Fig. S3, Supplementary Information) and consistent CIE-y values (~0.046) (Fig. 1g), demonstrating excellent long-term optical stability. Moreover, these enhanced PL properties were retained in solid films, as the target NPL films exhibited a PL QY of 70%, representing more than 10-fold enhancement over the control NPL films (6%) (Supplementary Fig. S4, Supplementary Information). Furthermore, the target NPL films demonstrate exceptional spectral stability, with no significant changes detected in the PL spectra after 60 days, and the CIE-y value remained stable at 0.046 (Supplementary Fig. S5, Supplementary Information). These results clearly show that the acid-assisted ligand passivation strategy significantly enhances the storage and spectral stability CsPbBr_3_ NPLs.

### Morphology and surface microstructure of CsPbBr_3_ NPLs

**Fig. 2:**
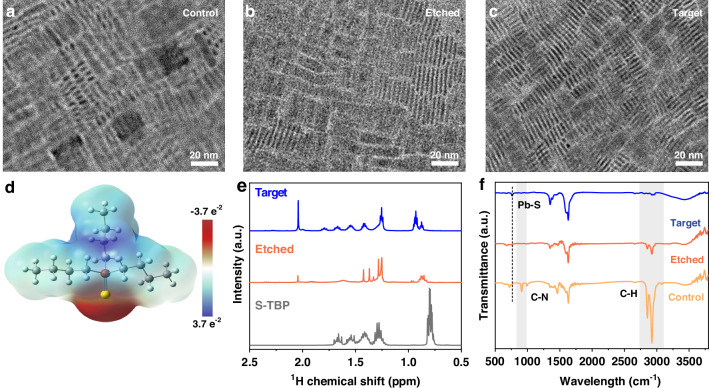
Structure and surface chemical characterization of CsPbBr_3_ NPLs. TEM images of **a** the control, **b** etched, and **c** target NPLs. **d** Electrostatic potential distribution of S-TBP ligand. **e**^1^H NMR spectra of S-TBP, etched, and target NPLs. **f** FTIR results of control, etched, and target NPLs

To investigate the morphological effects of the acid-assisted ligand passivation strategy on CsPbBr_3_ NPLs, transmission electron microscopy (TEM) characterization was performed. The TEM image shows that the control NPLs exhibit an uneven lateral size distribution (Fig. 2a). After HBr etching, the lateral size of the CsPbBr_3_ NPLs is significantly reduced, and the size distribution becomes more uniform (Fig. 2b). These morphological changes are consistent with the observed blue shift and narrowed emission linewidth in the PL spectra. Notably, the subsequent introduction of the short-chain ligand S-TBP does not induce any substantial changes in morphology or dimensions (Fig. 2c). X-ray diffraction (XRD) analysis further confirms that the crystal structure of the NPLs remains stable, with no observable peak shifts (Supplementary Fig. S6, Supplementary Information). These results indicate that the acid-assisted ligand passivation strategy enables efficient in situ ligand exchange while preserving the structural integrity and quantum confinement properties of CsPbBr_3_ NPLs.

To elucidate the surface chemical regulation mechanism of the acid-assisted ligand passivation strategy on CsPbBr_3_ NPLs, we systematically analyzed their surface composition using multiple characterization techniques. X-ray photoelectron spectroscopy (XPS) reveals characteristic S 2p (166-172 eV) and P 2p (128-135 eV) peaks in the target samples (Supplementary Fig. S7, Supplementary Information), directly confirming the successful anchoring of S-TBP ligands on the surface of CsPbBr_3_ NPLs. The electrostatic potential distribution of the S-TBP ligand, obtained through density functional theory (DFT) calculations, is shown in Fig. 2d. In this map, red regions indicate negative electrostatic potential, while blue regions denote positive potential. The localization of negative charge around the S atoms suggests a strong coordination tendency with surface Pb^2+^ ions. Comparative XPS analysis further reveals binding energy shifts for Pb 4f and Br 3d orbitals toward higher energies, along with a notable reduction in metallic Pb^0^ signals (Supplementary Fig. S8, Supplementary Information). These observations collectively demonstrate the effective reconstruction of the surface coordination environment of CsPbBr_3_ NPLs through the synergistic interaction of HBr etching and S-TBP ligand passivation.

To investigate the surface reconstruction mechanism of CsPbBr_3_ NPLs induced by acid-assisted ligand passivation, we performed a series of nuclear magnetic resonance (NMR) and Fourier transform infrared (FTIR) characterizations. The ^1^H NMR spectrum of the etched CsPbBr_3_ NPLs showed markedly reduced signal intensities in the 4.5-6.0 ppm range, corresponding to OAm ligands (Supplementary Fig. S9, Supplementary Information)^38^, indicating effective removal of native long-chain surface ligands. Notably, a new characteristic peak appeared in the 1-2 ppm region (Fig. 2e), with a chemical shift consistent with that of free S-TBP^39^, confirming its successful coordination to the NPL surface. Complementary^31^P NMR analysis revealed a chemical shift near 1 ppm within the 49-52 ppm region (Supplementary Fig. S10, Supplementary Information), suggesting partial cleavage of the S=P bond in S-TBP, with P remaining partially coordinated to S. These observations were further supported by FTIR measurements, where the etched sample exhibited pronounced attenuation of the C-N stretching vibration at 910 cm^-1^ and the olefinic C-H stretching bands at 2857.1-2925.8 cm^-1^ (Fig. 2f), indicating ligand removal. Concurrently, a new vibrational band at 766 cm^-1^ emerged in the target sample, attributable to the stretching vibration of the Pb-S bond^40,41^, in alignment with NMR evidence. Collectively, these results demonstrate that acid-assisted ligand passivation facilitates proton-mediated exfoliation of bulky organic ligands and enables the formation of stable Pb-S-P coordination bonds between S-TBP and surface Pb^2+^ ions, thereby significantly improving the surface passivation efficacy and structural integrity of CsPbBr_3_ NPLs.

**Fig. 3:**
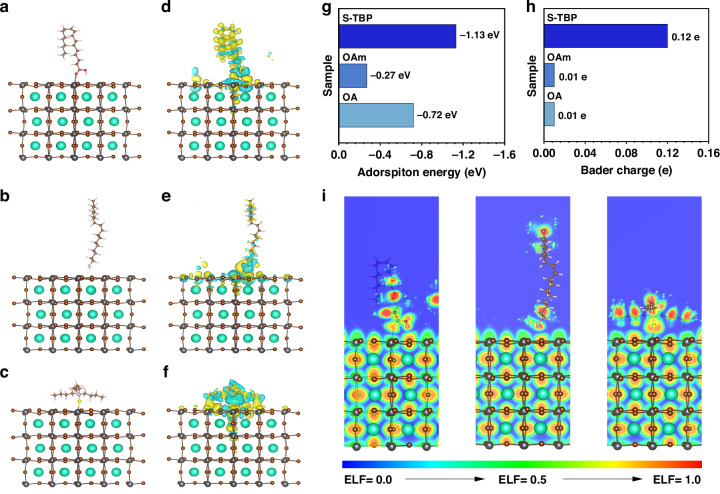
Theoretical investigation of ligands-NPLs interactions. Adsorption models of CsPbBr_3_ NPLs capped with **a** OA, **b** OAm, and **c** S-TBP ligands. Differential charge density distributions of NPLs capped with **d** OA, **e** OAm, and **f** S-TBP. **g** Comparison of adsorption energies for different ligands on NPL surfaces. **h** Bader charge analysis showing charge transfer from ligands to NPLs. **i** Electron localization function maps of NPLs passivated with different ligands

To elucidate the interactions between ligands and CsPbBr_3_ NPLs, DFT calculations (Supplementary Note S3, Supplementary Information) were performed using a PbBr_2_-terminated surface model (Fig. 3a–c). The calculated adsorption energies (E_ads_) reveal that the S atom in S-TBP exhibits a strong binding affinity to undercoordinated surface Pb^2+^ ions, with an E_ads_ of -1.13 eV. This is significantly higher than those of OA (-COO^-^ group, E_ads_ = -0.72 eV) and OAm (-N(CH_3_)_3_^+^ group, E_ads_ = -0.27 eV) (Fig. 3g), highlighting the superior passivation capability of S-TBP for surface dangling bond defects. Furthermore, differential charge density analyses of the optimized NPL-ligand systems (Fig. 3d–f) reveal that S-TBP induces more substantial interfacial charge redistribution compared to OA and OAm, thereby facilitating stronger electronic coupling in solid-state NPL films.

Bader charge analysis was performed to evaluate the extent of charge transfer between CsPbBr_3_ NPLs and various ligands, including OA, OAm, and S-TBP. The results show minimal charge transfer for OA and OAm (both 0.01e), whereas S-TBP exhibits a significantly higher charge transfer of 0.12e, an order of magnitude greater (Fig. 3h). This substantial increase directly supports the enhanced charge carrier transport observed in S-TBP-treated NPL solid-state films. Furthermore, electronic localization function analyses (Fig. 3i) provide atomic-level insights into ligand-NPL interactions. While none of the ligands induced noticeable structural changes on the NPL surface, S-TBP binding demonstrated a markedly stronger orbital overlap between Pb and S atoms compared to OA and OAm, indicating enhanced electronic coupling. These results disclose the superior surface passivation and interfacial charge transfer capabilities of S-TBP, which together contribute to improved stability and electronic performance of CsPbBr_3_ NPL films.

### Defect density of CsPbBr_3_ NPLs

**Fig. 4:**
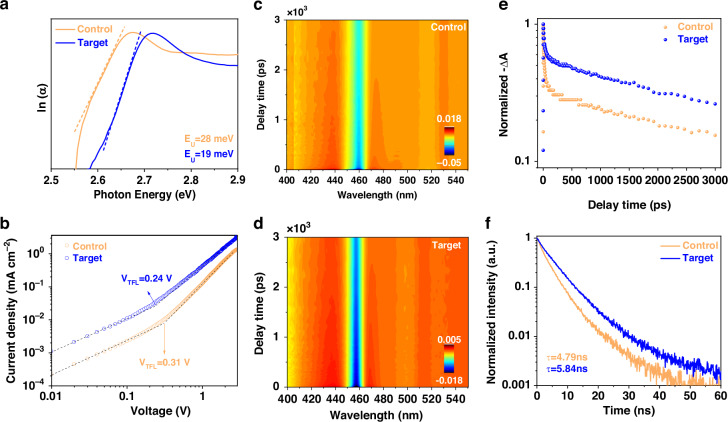
Defect density and recombination dynamics of CsPbBr_3_ NPLs. **a** Urbach energy plots of control and target CsPbBr_3_ NPLs. **b** Current density-voltage curves of control and target CsPbBr_3_ NPLs hole-only devices under dark conditions. TA spectroscopy of **c** control and **d** target CsPbBr_3_ NPLs. **e** Ground-state bleaching recovery kinetics for control and target CsPbBr_3_ NPLs. **f** PL decay curves of control and target CsPbBr_3_ NPLs

The wavelength asymmetry behavior observed in strongly quantum-confined CsPbBr_3_ NPLs is suppressed upon ligand passivation, indicating a significant reduction in defect states^42^. To quantitatively assess the change in defect density, the Urbach energy (*E*_*U*_) of various samples was calculated by plotting the absorption as a function of photon energy^43^ (Supplementary Note S4, Supplementary Information). As shown in Fig. 4a, control CsPbBr_3_ NPLs exhibit an *E*_*U*_ value of 28 meV, while the ligand-treated target NPLs demonstrate a reduced value of 19 meV, confirming that HBr-assisted surface reconstruction and S-TBP ligand binding effectively reduce surface defect density.

For a more precise quantification of defect density, the space-charge-limited current (SCLC) method was employed using hole-only carrier devices with an indium tin oxide (ITO)/poly(3,4-ethylenedioxythiophene) polystyrene sulfonate (PEDOT:PSS)/poly[bis(4-phenyl)(2,4,6-trimethylphenyl)amine] (PTAA)/CsPbBr_3_ NPLs/4,4’,4”-Tris(carbazol-9-yl)-triphenylamine (TCTA)/Ag architecture (Fig. 4b). The trap densities were determined using the equation^44^: ${N}_{t}=(2\varepsilon {\varepsilon }_{0}{V}_{{TFL}})/{({eL}}^{2})$, where *ε* and *ε*_*0*_ represent the dielectric constant and vacuum dielectric constant, *V*_*TFL*_ is the trap-filling limit voltage in the hole-only carrier device, *e* is the elementary charge, and *L* is the thickness of the CsPbBr_3_ NPL film (30 nm). The measured *V*_*TFL*_ values were 0.31 V for the control sample and 0.24 V for the target, yielding trap densities of 5.47 × 10^18 ^cm^-3^ and 4.23 × 10^18 ^cm^-3^, respectively. These results confirm that the acid-assisted passivation using S-TBP ligands significantly reduces trap density in CsPbBr_3_ NPLs, thereby improving their optical properties.

To investigate the influence of acid-assisted ligand passivation on the excited-state dynamics of CsPbBr_3_ NPLs, transient absorption (TA) and time-resolved PL measurements were performed. Both the control and target NPLs exhibited ground-state bleaching (GSB) signals centered at 460 nm, corresponding to the 1 s exciton transition, with signal intensities progressively decreasing due to exciton relaxation (Fig. 4c, d). Compared to the control NPLs, which displayed a broader GSB band (~20 nm), the target NPLs showed a narrower band (~13 nm) (Supplementary Fig. S11, Supplementary Information), indicating improved spectral symmetry as a result of ligand passivation. The GSB recovery kinetics further revealed a slower decay in the passivated NPLs, suggesting suppressed non-radiative recombination pathways. Quantitative analysis of the TA spectra showed prolonged lifetimes across all decay components in the target sample (Supplementary Table S1, Supplementary Information), implying effective suppression of exciton trapping and enhanced photophysical stability^45^. This result was further confirmed by the time-resolved PL measurements (Supplementary Note S5, Supplementary Information), which showed that the average PL lifetimes increased from 4.79 ns in the control to 5.84 ns in the target NPLs. Calculated radiative (*k*_*r*_) and non-radiative (*k*_*nr*_) recombination rates (Supplementary Note S6, Supplementary Table S2, Supplementary Information) showed a 4-fold increase in *k*_*r*_ and a 25-fold decrease in *k*_*nr*_ for the target NPLs compared to the control NPLs, indicating low defect density and reduced non-radiative recombination losses. Upon photoexcitation, electrons in CsPbBr_3_ NPLs are promoted from the valence band to the conduction band, generating free excitons (Supplementary Fig. S12, Supplementary Information). Collectively, these results demonstrate that acid-assisted ligand passivation effectively mitigates surface defects caused by incomplete octahedral coordination and bromine vacancies. As a result, the exciton trapping probability is reduced, leading to significant improvements in the PL performance of CsPbBr_3_ NPLs.

### Electroluminescent performance of CsPbBr_3_ NPL based PeLEDs

**Fig. 5:**
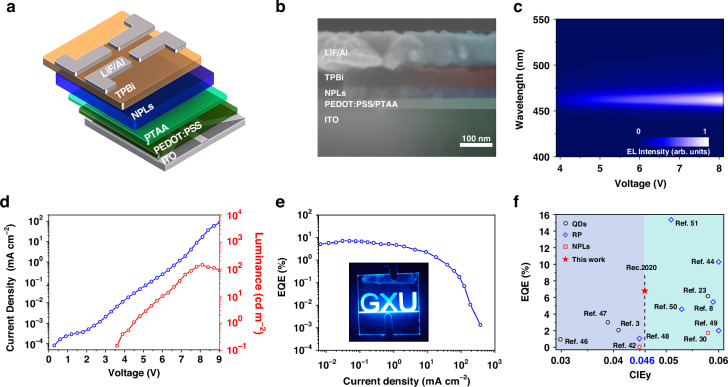
EL performance of CsPbBr_3_ NPL based PeLEDs. **a** Schematic illustration of the device architecture. **b** Cross-sectional SEM image of the PeLED. **c** Two-dimensional plot of the EL spectra under varying driving voltages. **d** Current density-voltage-luminance characteristics. **e** EQE versus current density for PeLEDs. The inset shows a photo of the PeLED at a voltage of 7.5 V. **f** Comparison of EQE versus CIE-y values for representative blue PeLEDs^3,8,23,30,42,44,46–53^

To evaluate the application potential of CsPbBr_3_ NPLs in deep-blue light-emitting devices, we fabricated PeLEDs using both control and target NPLs. The device structure, as shown in Fig. 5a, consists of ITO/PEDOT:PSS/PTAA (35 nm)/CsPbBr_3_ NPLs (30 nm)/1,3,5-tris(1-phenyl-lh-benzomidazol-2yl) benzene (TPBi, 50 nm)/ lithium fluoride (LiF, 1 nm)/ aluminum (Al, 100 nm), with corresponding cross-sectional SEM images presented in Fig. 5b. PeLEDs based on control NPLs exhibited an electroluminescence (EL) peak at 476 nm (Supplementary Fig. S13, Supplementary Information), which red-shifted under increasing voltage due to electric field-induced NPL aggregation. In contrast, PeLEDs employing target NPLs displayed a sharp EL peak at 461 nm with a narrow FWHM of 13 nm (Fig. 5c) and excellent spectral stability under varying voltages (Supplementary Fig. S14, Supplementary Information). The CIE coordinates of the target-NPL-based PeLEDs were (0.136, 0.046), fully meeting the Rec.2020 standard for deep-blue emission (Supplementary Fig. S15, Supplementary Information). Additionally, these devices achieved a maximum brightness of 143 cd m^-2^ and an EQE of 6.81% (Fig. 5d, e), far surpassing the 8 cd m^-2^ brightness and 0.07% EQE of the control devices (Supplementary Fig. S16, Supplementary Information). This performance, representing the highest reported EQE for PeLEDs with NPL-based (Supplementary Table S3, Supplementary Information) and CIEy ≤ 0.046, is comparable to the most state-of-the-art pure-blue PeLEDs (Fig. 5f), suggesting the effectiveness of the acid-assisted ligand passivation strategy in realizing high-performance deep-blue PeLEDs.

The excellent device performance can be attributed to the improved film quality and optoelectronic properties enabled by passivation. The target NPL films exhibited smoother morphology with reduced root-mean-square (RMS) roughness (from 4.27 nm to 2.58 nm, Supplementary Fig. S17, supporting information), which helps minimize surface defects and facilitates efficient charge transport. PL mapping further confirmed more uniform and intense emission across the film (Supplementary Fig. S18), indicating suppressed non-radiative recombination. Moreover, the electrical conductivity of the target NPL films was enhanced by a factor of two compared to the control (1.00 × 10^-4^ vs. 5.25 × 10^-5^ S m^-^^1^, Supplementary Fig. S19, Supplementary Note S7, Supporting Information), while electrochemical impedance spectroscopy revealed a substantial reduction in recombination resistance (from 62 kΩ to 9.8 kΩ, Supplementary Fig. S20, Supporting Information), reflecting more efficient carrier recombination. Together, these improvements in film morphology, luminescence uniformity, conductivity, and charge recombination dynamics underpin the superior performance of the deep-blue PeLEDs based on acid-passivated CsPbBr_3_ NPLs.

## Discussion

In summary, we have developed efficient deep-blue PeLEDs based on colloidal CsPbBr_3_ NPLs that meet the stringent Rec.2020 color standard, enabled by an acid-assisted short-chain ligand passivation strategy. Both theoretical calculations and experimental results demonstrate that the short-chain ligand S-TBP anchors strongly to the surface of CsPbBr_3_ NPLs via robust Pb-S-P coordination, significantly enhancing environmental stability while preserving the intrinsic quantum confinement effect. Compared to conventional long-chain ligands OA and OAm, S-TBP exhibits a higher adsorption energy (E_ads_ = -1.13 eV), forming a more stable passivation layer. The passivated CsPbBr_3_ NPLs maintain stable CIE chromaticity coordinates in both colloidal solution and solid-state film under ambient conditions, while effectively suppressing trap-mediated recombination. As a result, the optimized PeLEDs achieve a high EQE of 6.81%, a peak luminance of 143 cd m^-2^, and a deep-blue CIE-y coordinate of 0.046, fully compliant with the Rec.2020 standard. This work presents an effective strategy for the development of high-efficiency, stable deep-blue PeLEDs, demonstrating strong potential for the commercialization of perovskite in next-generation ultra-high-definition display technologies.

## Materials and methods

### Materials

Cesium carbonate (Cs_2_CO_3_, 99.99%), oleic acid (OA, 85%), 1-octadecene (ODE, 90%), lead bromide (PbBr_2_, 99.99%), oleylamine (OAm, 80-90%), hydrogen bromide (HBr, 40 wt.% in H_2_O), sulfur (S, 99.999%), tributyl phosphine (TBP, 95%), and ethyl acetate (EA, 99.9%) were acquired from Aladdin. Hexane (99%), LiF (99.99%), and chlorobenzene (CB, 99.8%) were purchased from Innochem. Toluene (99.8%) was obtained from Nanning Lantian Experimental Equipment Co., Ltd. PEDOT:PSS (Al 4083, Heraeus), and TPBi purchased from Volt-Amp Optoelectronics Tech. Co., Ltd. PTAA was acquired from Xi’an Yuri Solar Co., Ltd.

### Preparation of Cs-oleate

0.2 g Cs_2_CO_3_, 7 mL ODE, and 0.6 mL OA were introduced into three-necked flask. Under vacuum, the solution was heated to 120 °C and maintained at this temperature for 30 min. Subsequently, under the N_2_ atmosphere, temperature was raised to 150 °C until the Cs_2_CO_3_ powder was completely dissolved, forming Cs-oleate (Cs-OA).

### Preparation of control CsPbBr_3_ NPLs

0.07 g PbBr_2_ and 5 mL ODE were introduced into three-necked flask. Vacuum drying at ambient temperature for 30 min and injection of 0.5 mL OA and 0.5 mL OAm to achieve complete dissolution of the PbBr_2_ precursor. Following solvent degassing, the atmosphere was replaced with N_2_, and the solution was gradually elevated to 100 °C under continuous stirring. This preheated solution was then rapidly injected into the reaction. Subsequent rapid quenching using an ice-water bath facilitated the growth of control CsPbBr_3_ NPLs.

### Preparation of S-TBP

0.24 g S and 5 mL TBP were introduced into three-necked flask. The solution was dried under vacuum at 120 °C for 40 min and subsequently cooled to 30 °C under N_2_ atmosphere to form S-TBP.

### Preparation of HBr etching CsPbBr_3_ NPLs

0.07 g PbBr_2_ and 5 mL ODE were introduced into three-necked flask. Vacuum drying at ambient temperature for 30 min and injection of 0.5 mL OA and 0.5 mL OAm to achieve complete dissolution of the PbBr_2_ precursor. Following solvent degassing, the atmosphere was replaced with nitrogen, and the solution was gradually elevated to 100 °C under continuous stirring. This preheated solution was then rapidly injected into the three-necked flask. Subsequently, the mixture was immediately cooled to 60 °C, HBr was quickly injected, and then the mixture was then cooled quickly to 30 °C.

### Preparation of target CsPbBr_3_ NPLs

The process is identical for etched and target NPLs except for ligand exchange. After injection of HBr, the S-TBP solution is added. The mixture was then cooled quickly.

### Purification

Add 15 mL of EA to the reaction and centrifuge at 10,000 rpm for 3 min. Then precipitate was dispersed in 5 mL of a 1/2 mixture of toluene and EA and after centrifugation at 10,000 rpm for 3 min, the precipitate was redispersed in 1 mL of hexane to further use.

### Device fabrication

Filtered PEDOT: PSS was spin-coated onto the UV-ozone-treated ITO substrate at 5000 rpm and then annealed in air at 150 °C for 20 min. Next, PTAA dispersed in CB was spin-coated onto the surface at 2000 rpm and annealed at 120 °C in a nitrogen atmosphere. Subsequently, an octane solution of CsPbBr_3_ NPLs was spin-coated at 2000 rpm. Finally, TPBi, LiF, and Al were sequentially deposited via thermal evaporation under vacuum.

### Characterization

Steady-state PL spectra of CsPbBr_3_ NPL colloidal solutions and films were acquired on a Fluorolog-3 spectrofluorometer. TRPL were measured using a 368 nm pulsed laser. UV-visible absorption spectra of CsPbBr_3_ NPLs were recorded using a Shimadzu UV-2700 spectrophotometer. PL QYs were determined using a Hamamatsu Photonics Quantaurus-QY (C11347) system under 365 nm excitation. TEM images were obtained on an FEI TECNAI G2 F30 microscope. XPS analysis was enforced using an ESCALAB 250Xi equipped with monochromatic Al K*α* radiation. XRD analysis was performed using an a Rigaku D/MAX 2500 V diffractometer with Cu Kα radiation (λ = 1.540 Å). fs-TA spectroscopy was conducted using a femto-TA100 spectrometer (Time-Tech Spectra, China) equipped with a fiber laser (1030 nm, 100 kHz repetition rate, 9.5 μJ/pulse, YF-FL-10-100-IR, Yacto-Technology). NMR spectra were recorded on a Zhongke Niujin AS400 spectrometer. FTIR spectra were measured using a Shimadzu IRTracer-100 spectrometer. SEM cross-sectional images were acquired on a Hitachi SU8000 field-emission microscope. AFM measurements were performed on a Bruker Dimension Icon system in tapping mode. Electrical performance was evaluated using a Keithley 2400 source meter equipped with an XPQY-EQE system (Guangzhou Xipu Optoelectronics Technology Co., Ltd.).

## Supplementary information

Supplementary Information

## Data Availability

The data that support the findings of this study are available from the corresponding author upon reasonable request.
