# Supplementary material for: Efficient deep-blue LEDs based on colloidal CsPbBr3 nanoplatelets meeting the Rec.2020 standard
Source: Light Sci Appl. 2025 Sep 22;14:336. doi: 10.1038/s41377-025-02019-1 (PMC12454636; doi:10.1038/s41377-025-02019-1)
Supplement: Supplementary file 1 — Supplementary Information [file 41377_2025_2019_MOESM1_ESM.docx]

**Supplementary Information for**

**Efficient deep-blue LEDs based on colloidal CsPbBr_3_ nanoplatelets meeting the Rec.2020 standard**

Yusheng Song^1^, Sheng Cao^1^*, Yijie Wang^1^, Mingyan Chen^1^, Yu Zhang^2^, Qiuyan Li^1^, Shulin Han^3^, Yi Liang^1^, Lei Cai^3^, Jialong Zhao^1^*, Bingsuo Zou^1,4^*

^1^School of Physical Science and Technology, State Key Laboratory of Featured Metal Materials and Life-cycle Safety for Composite Structures, Guangxi University, Nanning 530004, China.

^2^Hubei Key Laboratory of Energy Storage and Power Battery, School of Optoelectronic Engineering, School of New Energy, Hubei University of Automotive Technology, Shiyan 442002, China

^3^Shandong Province Key Laboratory of Medical Physics and Image Processing Technology, School of Physics and Electronics, Institute of Materials and Clean Energy, Shandong Normal University, Jinan 250014, China.

^4^School of Resources, Environment and Materials, Guangxi University, Nanning 530004, China.

E-mails: caosheng@gxu.edu.cn; [zhaojl@gxu.edu.cn](mailto:zhaojl@gxu.edu.cn); [zoubs@gxu.edu.cn](mailto:zoubs@gxu.edu.cn).

**Note S1.**

For conventional OA and OAm capped NPLs, the equilibrium relationship exists between the ionization and molecular forms of these ligands after the introduction of HBr. As the following equations:

$$\begin{aligned} OAm+HBr\rightleftharpoons\mathrm{OAmH}^{+}+\mathrm{Br}^{-}\#(1) \end{aligned}$$

$$\begin{aligned} \mathrm{OAmH}^{+}+\mathrm{OA}^{-}\rightleftharpoons OAm+OAH\#(2) \end{aligned}$$

**Note S2.**

The color purity is the degree to which a color mixes with other colors and is usually defined as the ratio of the chromatic distance from the white point (representing the reference white light) to a given color to the total distance from the white point to the desired color. The color purity is calculated using the following equation:

$$\begin{aligned} Color purity=\frac{\sqrt{\left( x_{s}-x_{i} \right)^{2}+\left( y_{s}-y_{i} \right)^{2}}}{\sqrt{\left( x_{d}-x_{i} \right)^{2}+\left( y_{d}-y_{i} \right)^{2}}}\#\left( 3 \right) \end{aligned}$$

where *x_s_* and *y_s_* are CIE color coordinates of the CsPbBr_3_ NPLs, *x_i_* and *y_i_* are the white point of the 1931 CIE standard source (*x_i_*, *y_i_*) = (0.3127, 0.3290), *x_d_* and *y_d_* are the color coordinates (0.131, 0.046) given in Rec.2020.


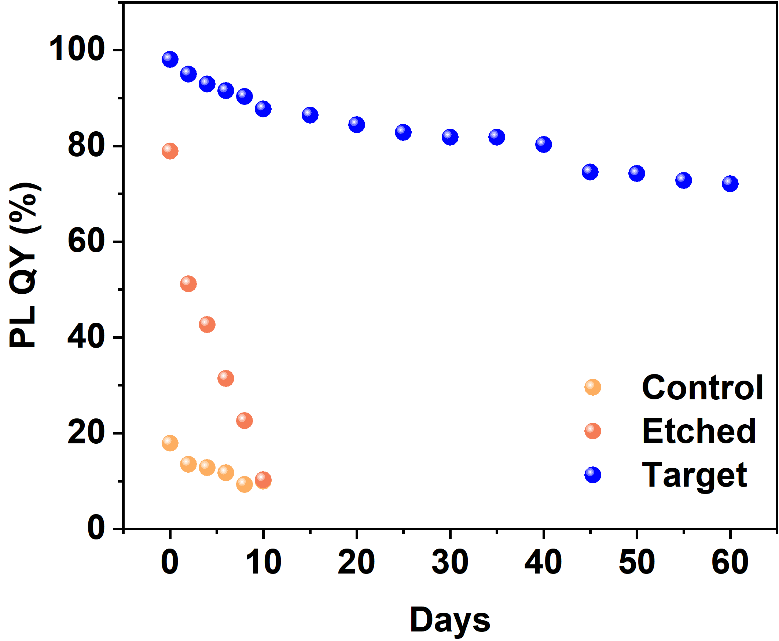


**Fig. S1.** Long-term PL QY stability of control, etched, and target CsPbBr_3_ NPL colloidal solutions under ambient conditions (25 ± 5 °C, 55 ± 20% RH).

As shown in Fig. S1, colloidal solution stability of the three samples was tested at a temperature of 25 ± 5 °C and a humidity of 55 ± 20% RH. After 10 days, PL QYs of both control and etched CsPbBr_3_ NPLs dropped below 50% of the original value. Therefore, PL QYs for control and etched NPLs after 10 days were not provided.


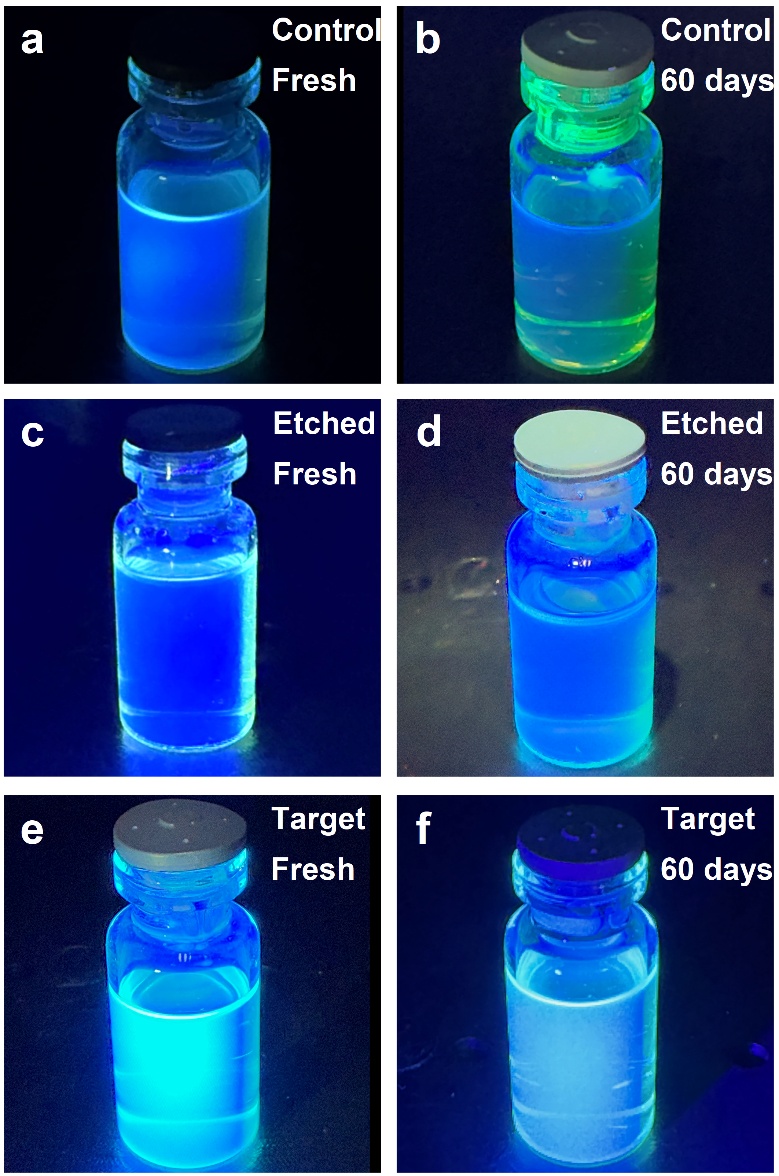


**Fig. S2.** Photos of fresh (a) control, (c) etched, and (e) target CsPbBr_3_ NPL colloid solutions and after 60 days, (b) control, (d) etched, and (f) target CsPbBr_3_ NPL colloid solutions under 365 nm UV lamp illumination.


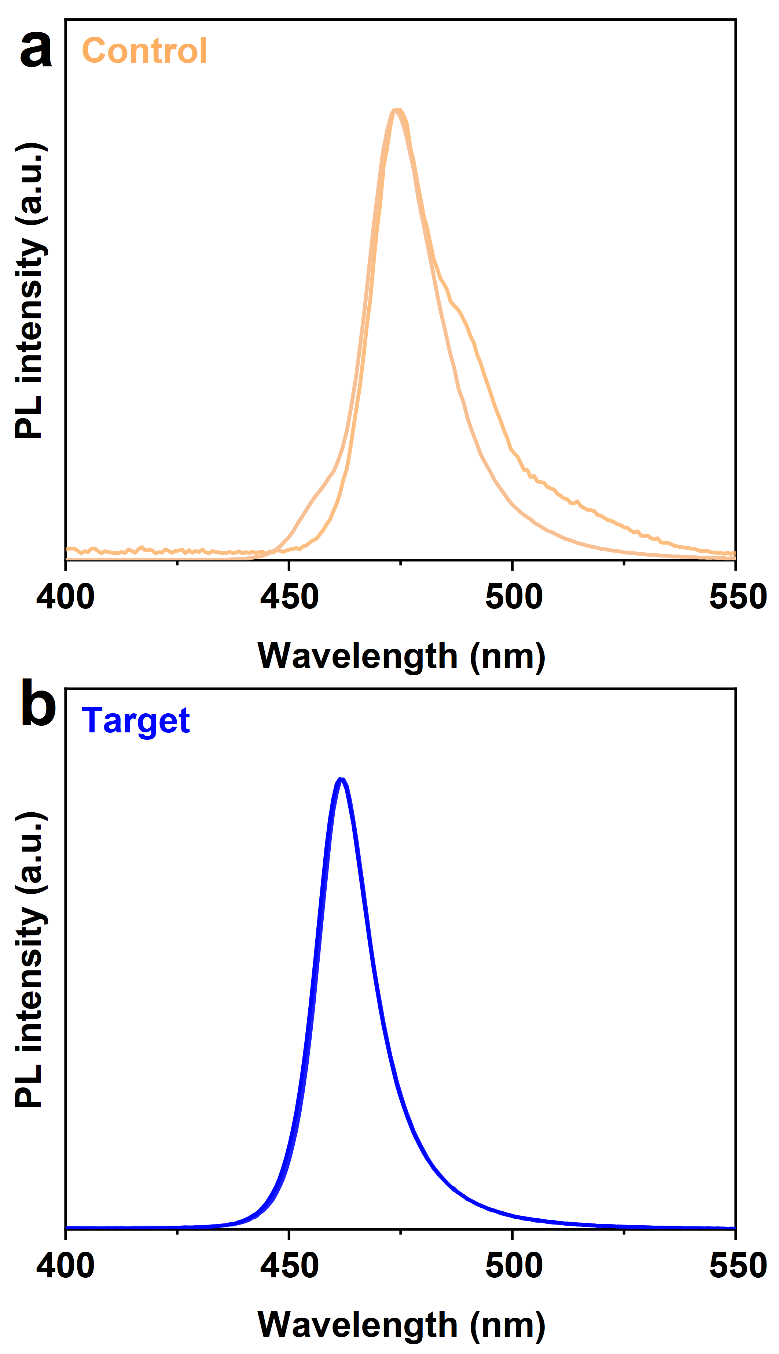


**Fig. S3.** PL spectra of (a) control and (b) target CsPbBr_3_ NPL colloidal solutions before and after 60 days.


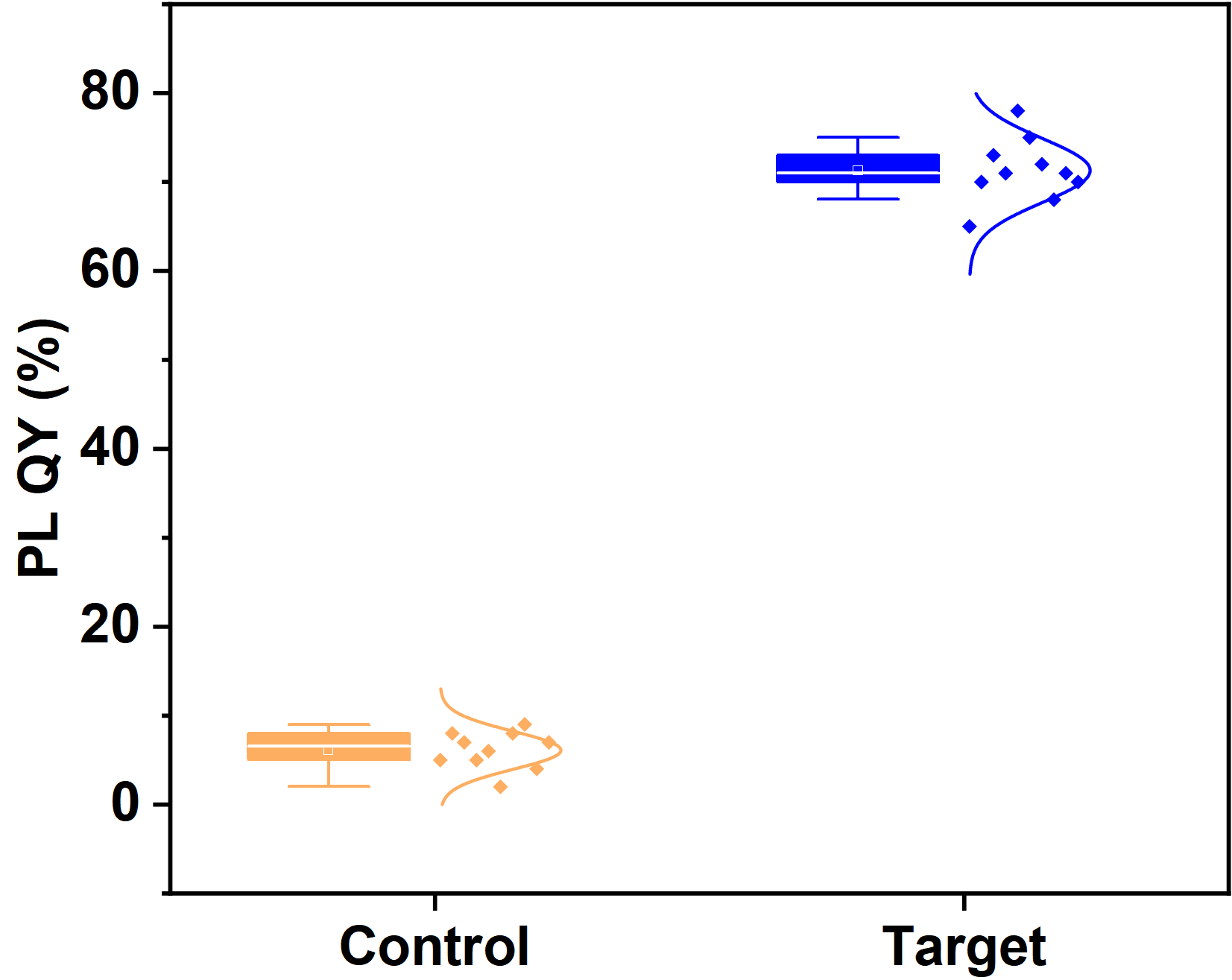


**Fig. S4.** Statistical distribution of PL QYs measured across 10 independent synthetic batches for control and target NPL films.


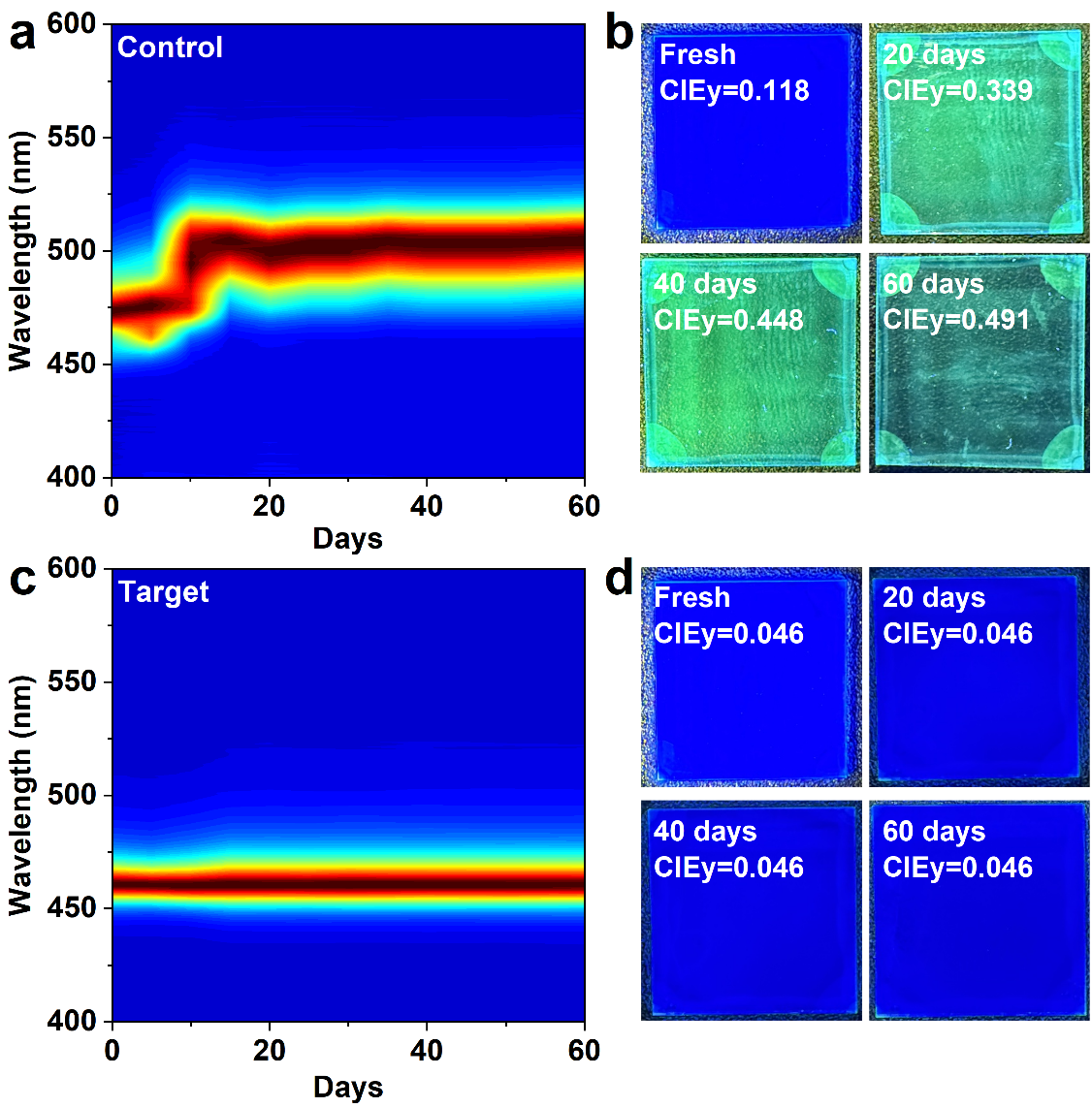


**Fig. S5.** 2D pseudo-color PL spectra of (a) control versus (c) target NPL films stored for 60 days at 25 ± 5 °C and 55 ± 20% RH. Photos of (b) control and (d) target NPL films stored for 60 days at 25 ± 5 °C and 55 ± 20% RH (recording interval of 20 days).

As shown in Figs. S5a and S5b, the PL peak of the control NPL film shifted from blue emission to green emission with time, CIE-y color coordinate increased from 0.118 to 0.491 and accompanied by a visible color change from blue to green under UV irradiation. While the target NPL films exhibited stable PL spectra and CIE-y (see Figs. S5c and S5d).


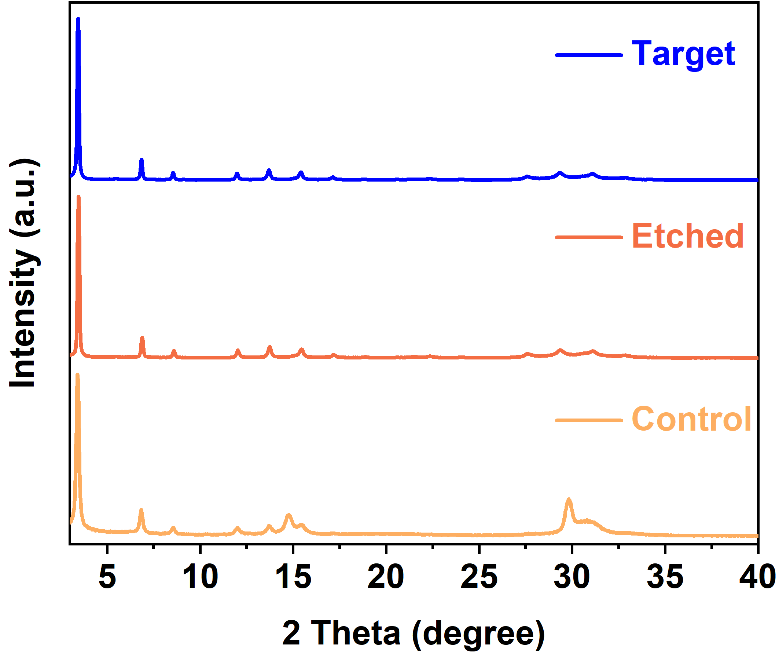


**Fig. S6.** XRD patterns of control, etched, and target CsPbBr_3_ NPLs.


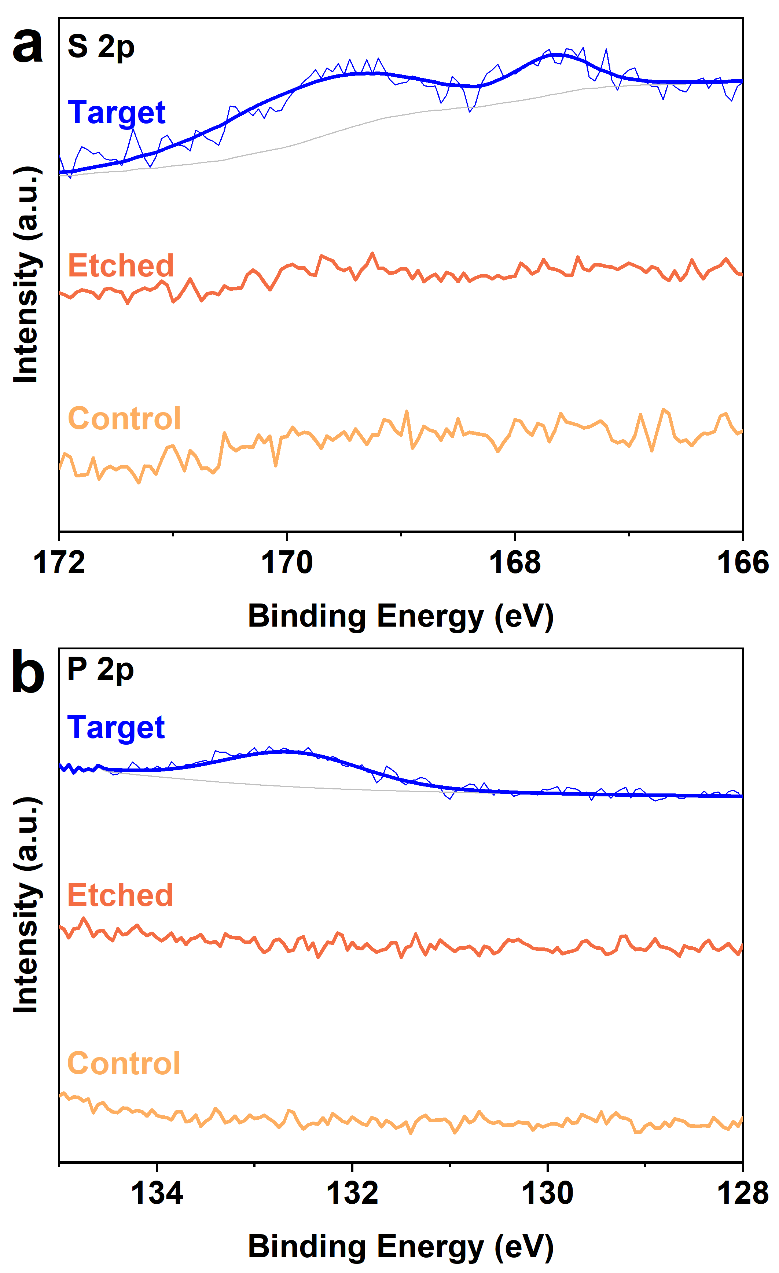


**Fig. S7.** (a) S 2p and (b) P 2p XPS spectra of control, etched, and target CsPbBr_3_ NPLs.


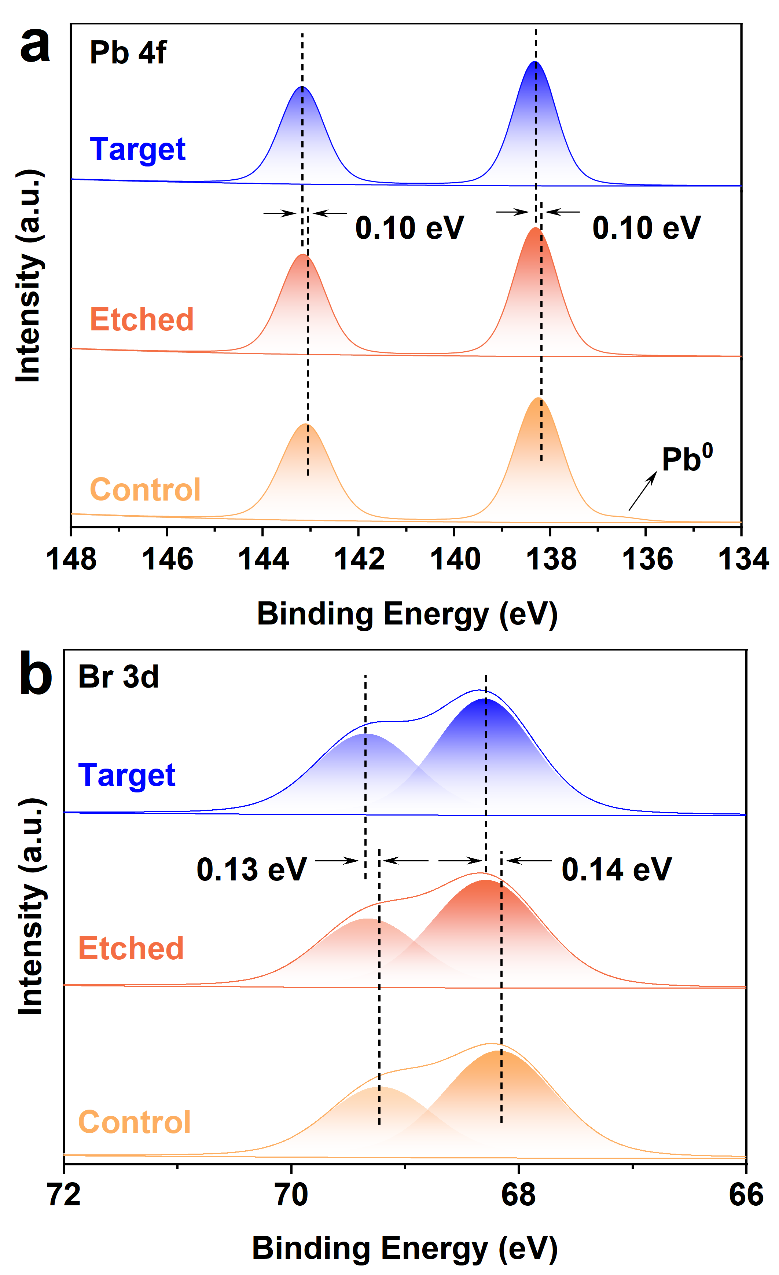


**Fig. S8.** (a) Pb 4f and (b) Br 3d XPS spectra of control, etched, and target CsPbBr_3_ NPLs.


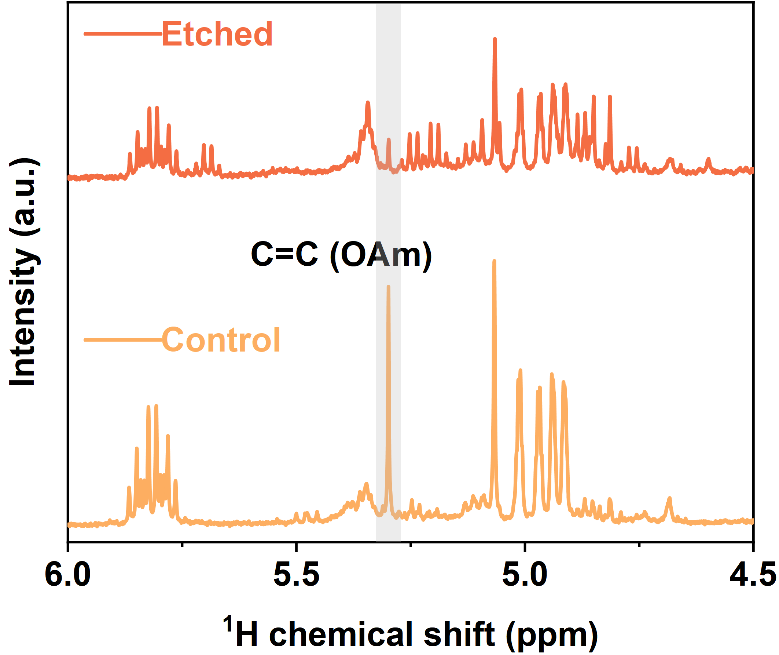


**Fig. S9.** ^1^H NMR spectra of control and etched CsPbBr_3_ NPLs.


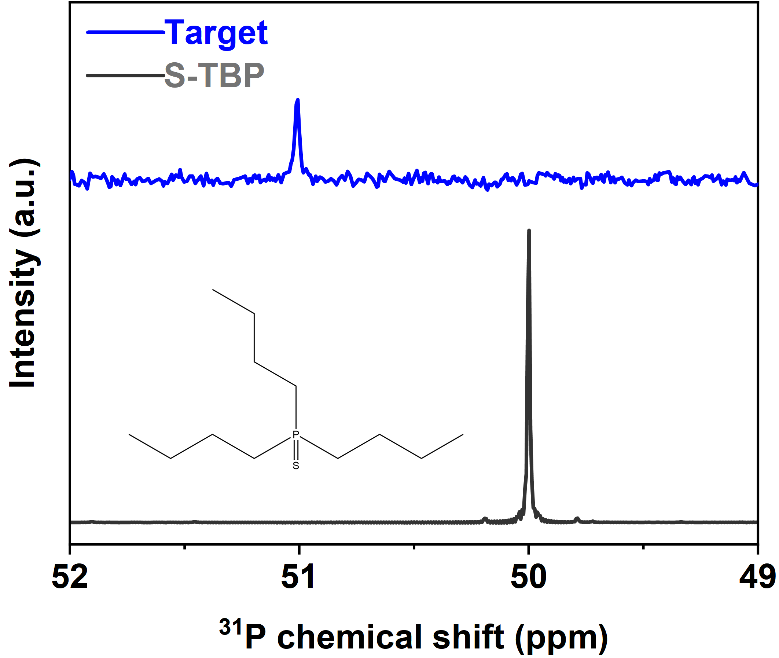


**Fig. S10.** ^31^P NMR spectra of S-TBP and target CsPbBr_3_ NPLs.

**Note S3.**

The Vienna Ab Initio Simulation Package performs density functional theory (DFT) calculations with the projector-augmented wave method^1^. The generalized gradient approximation is realized by the Perdew-Burke-Ernzerhof function with the projected augmented wave method and the plane-wave energy cutoff of 400 eV (ref. 2). The Monkhorst-Pack k-point grid is set as 4 × 4 × 4 for bulk structure optimization, and 4 × 4 × 1 for heterostructure. The DFT+D3 method is adopted for considering van der Waals (vdW) interaction^3^. The convergence criterion for Hellmann-Feynman forces and total energy are set as 0.01 eV Å^-1^ and 10^-5^ eV, and a vacuum slab larger than 25 Å is used to avoid the interaction between adjacent images.

All quantum chemical calculations are performed using Gaussian09. For the initial structure, the ligand molecule was optimized using the B3LYP density functional with the 6-311G (d, p) basis set for C, H, O, N, S, and P elements. Optimized geometries were verified by frequency calculations at the same level of theory as that used for geometry optimization.

For the perovskite crystal system with and without defect, under the same density functional and basis set, the adsorption energy was calculated by the following equation:

$$\begin{aligned} E_{ads}=E_{ligand molecule & perovskite}-\left（ E_{ligand molecule}+E_{perovskite} \right）\#\left( 4 \right) \end{aligned}$$

**Note S4.**

The Urbach energy can be extracted by fitting the exponential tail of the absorption coefficient according to the following equations:

$$\begin{aligned} \alpha\left( E \right)= \alpha_{0} \exp\left[ \sigma\left( T \right)\frac{E -E_{0}}{k_{B} T} \right]\#\left( 5 \right) \end{aligned}$$

$$\begin{aligned} E_{U}=\frac{k_{B} T}{\sigma\left( T \right)}\#\left( 6 \right) \end{aligned}$$

where *α(E)* is the absorption coefficient as a function of photon energy *E*, *E_0_* and *α_0_* are the characteristic parameters of the material, *σ(T)* is the steepness parameter, *k_B_* is the Boltzmann constant, and *T* is the thermodynamic temperature.


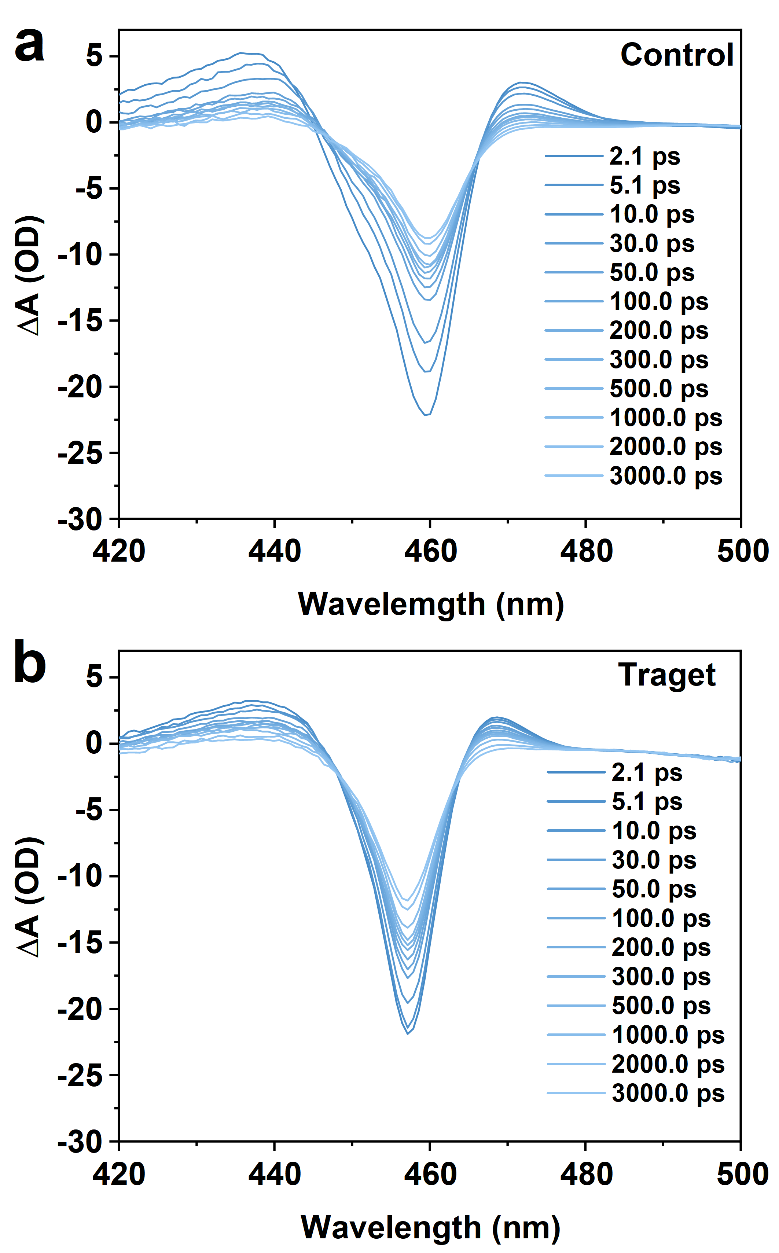


**Fig. S11.** TA spectra at different delay times of (a) control and (b) target CsPbBr_3_ NPLs.

**Table. S1** Parameters for the bleaching dynamics of CsPbBr_3_ NPLs.

| **Sample** | **A_1_** | **τ_1_ (ps)** | **A_2_** | **τ_2_ (ps)** | **A_3_** | **τ_3_ (ps)** |
| --- | --- | --- | --- | --- | --- | --- |
| **Control** | 0.45 | 3.69 | 0.36 | 24.09 | 0.19 | 1375.33 |
| **Target** | 0.31 | 10.05 | 0.24 | 46.17 | 0.45 | 2384.22 |

**Note S5.**

The PL decay curves of NPLs were fitted using a two-exponential function:

$$\begin{aligned} y=A_{1}\times exp\left( -\frac{i}{\tau_{1}} \right)+A_{2}\times exp\left( -\frac{i}{\tau_{2}} \right)\#(7) \end{aligned}$$

where *A_i_* and *τ_i_* represent the amplitude and decay time of each component, respectively.

The average exciton PL lifetime ($\tau_{ave}$) was then calculated by the following equation:

$$\begin{aligned} \tau_{ave}=\frac{A_{1}\times\tau_{1}^{2}+A_{2}\times\tau_{2}^{2}}{A_{1}\times\tau_{1}+A_{2}\times\tau_{2}}\#\left( 8 \right) \end{aligned}$$

**Note S6.**

The radiative (*k_r_*) and non-radiative (*k_nr_*) recombination rates based on the measured PL QY and average PL lifetime ($\tau_{ave}$) using the following equations:

$$\begin{aligned} k_{r}=\frac{\eta_{PL QY}}{\tau_{ave}}\#\left( 9 \right) \end{aligned}$$

$$\begin{aligned} k_{nr}=\frac{{1-\eta}_{PL QY}}{\tau_{ave}}\#\left( 10 \right) \end{aligned}$$

**Table. S2** The *k_r_* and *k_nr_* of the control and target CsPbBr_3_ NPLs.

| Sample | *k_r_* (×10^7^ s^-1^) | *k_nr_* (×10^7^ s^-1^) |
| --- | --- | --- |
| Control | 3.97 | 16.91 |
| Target | 16.44 | 0.68 |


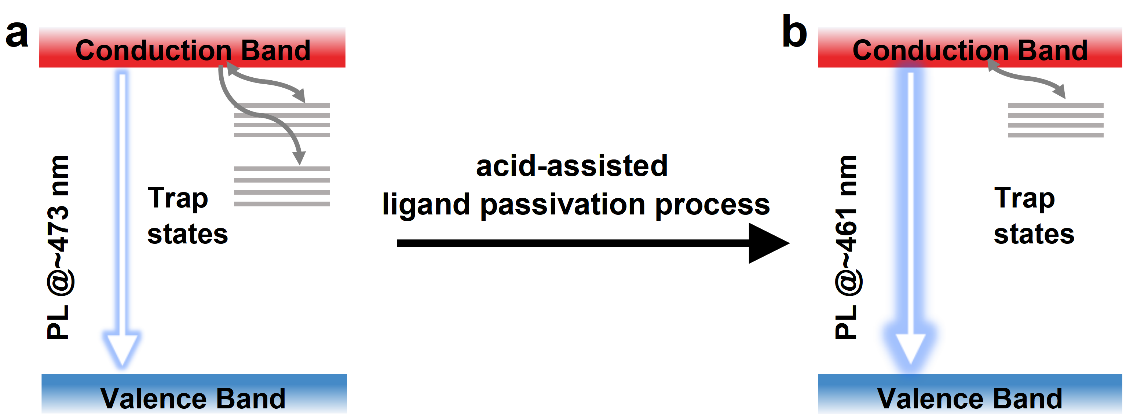


**Fig. S12.** Schematic representations of recombination dynamics for (a) control and (b) target CsPbBr_3_ NPLs.


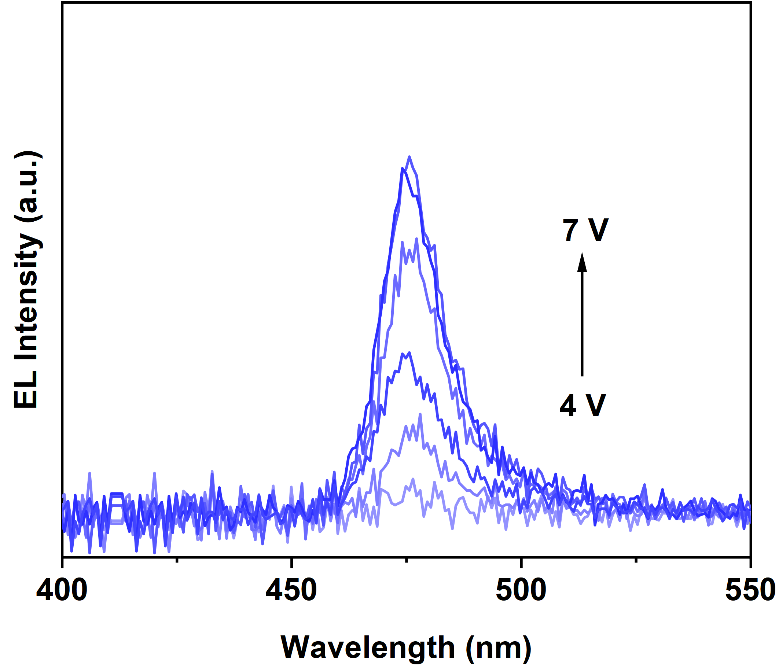


**Fig. S13.** Electroluminescence spectra of PeLED based on control CsPbBr_3_ NPLs under different voltages.


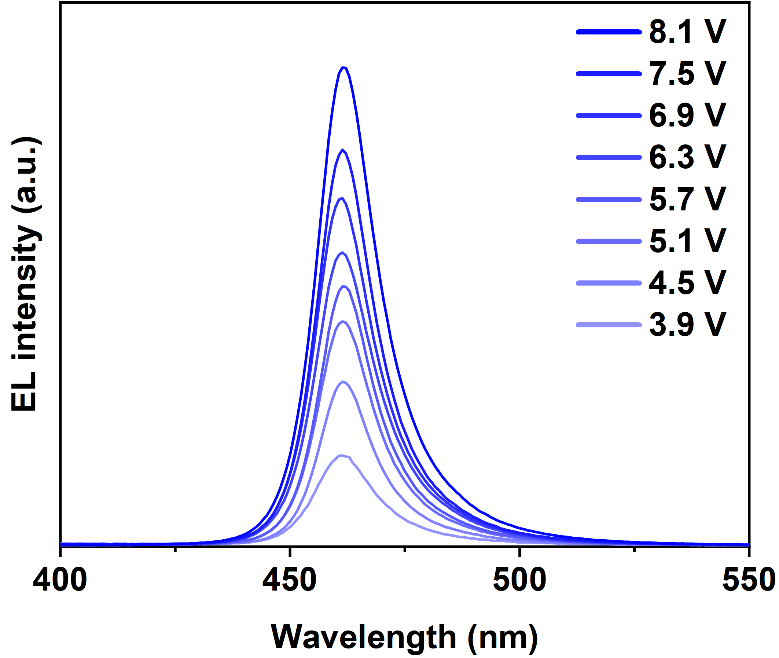


**Fig. S14.** Electroluminescence spectra of PeLED based on target CsPbBr_3_ NPLs under different voltages.


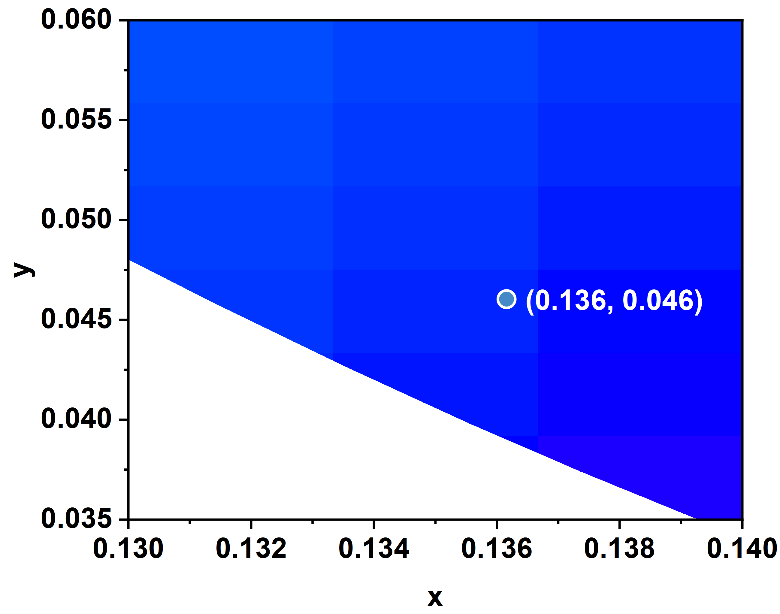


**Fig. S15.** The CIE coordinate of PeLED based on target CsPbBr_3_ NPLs at a voltage of 8.1 V.


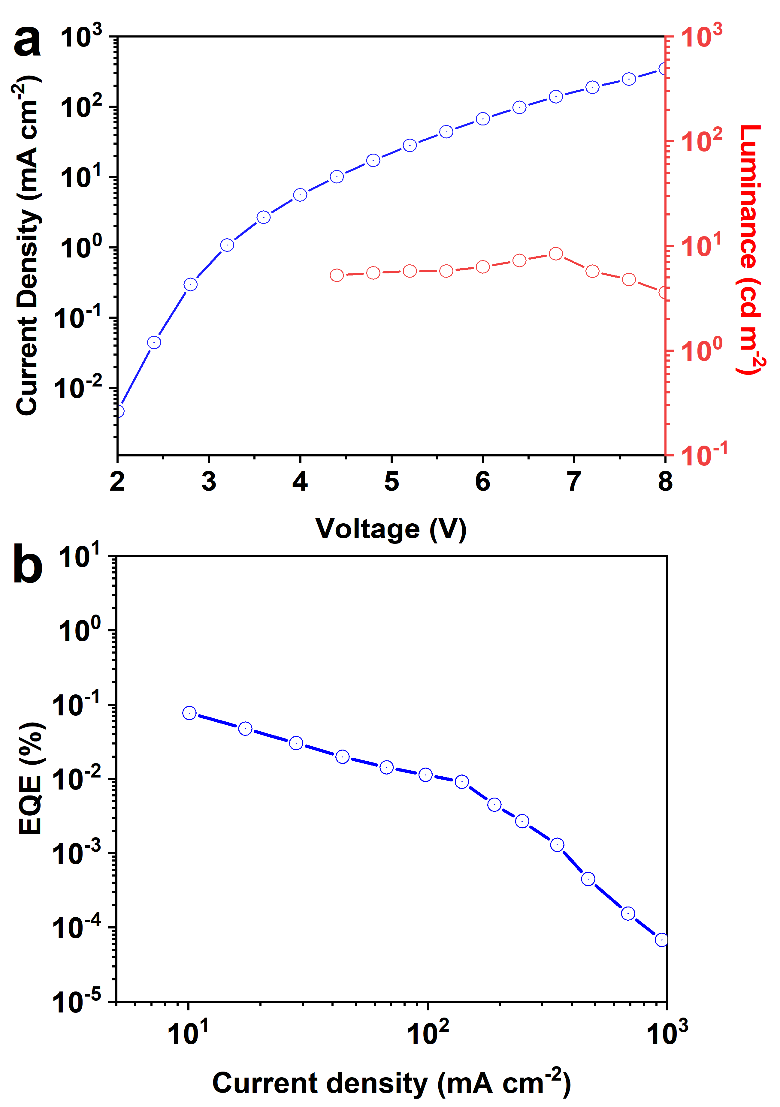


**Fig. S16.** (a) The current density-voltage-luminance curves and (b) EQE-current density characterization curves of PeLED based on control CsPbBr_3_ NPLs.

**Table. S3** Performance comparison of CsPbBr_3_ NPL-based PeLEDs in recent studies.

| **Emitting layer** | **Structural forms** | **EL peak (nm)** | **Luminance (cd m^-2^)** | **EQE (%)** | **CIE-y** | **Year** | **Ref.** |
| --- | --- | --- | --- | --- | --- | --- | --- |
| CsPbBr_3_ | 2D | 463 | 62 | 0.124 | 0.045 | 2018 | 4 |
| CsPbBr_3_ | 2D | 469 | 19.2 | 1.42 | NA | 2019 | 5 |
| CsPbBr_3_ | 2D | 465 | 631 | 0.8 | NA | 2021 | 6 |
| CsPbBr_3_ | 2D | 463 | 74 | 2 | 0.08 | 2022 | 7 |
| CsPbBr_3_ | 2D | 460 | 158.3 | 3.18 | 0.046 | 2022 | 8 |
| CsPbBr_3_ | 2D | 462 | 691 | 1.77 | 0.058 | 2023 | 9 |
| CsPbBr_3_ | 2D | 455 | 1511 | 4.15 | NA | 2023 | 10 |
| CsPbBr_3_ | 2D | 460 | 591 | 1.6 | 0.062 | 2024 | 11 |
| CsPbBr_3_ | 2D | 465 | 227 | 5.44 | 0.061 | 2024 | 12 |
| CsPbBr_3_ | 2D | 462 | NA | 4 | 0.055 | 2024 | 13 |
| CsPbBr_3_ | 2D | 462 | 143 | 6.81 | 0.046 | 2025 | This work |


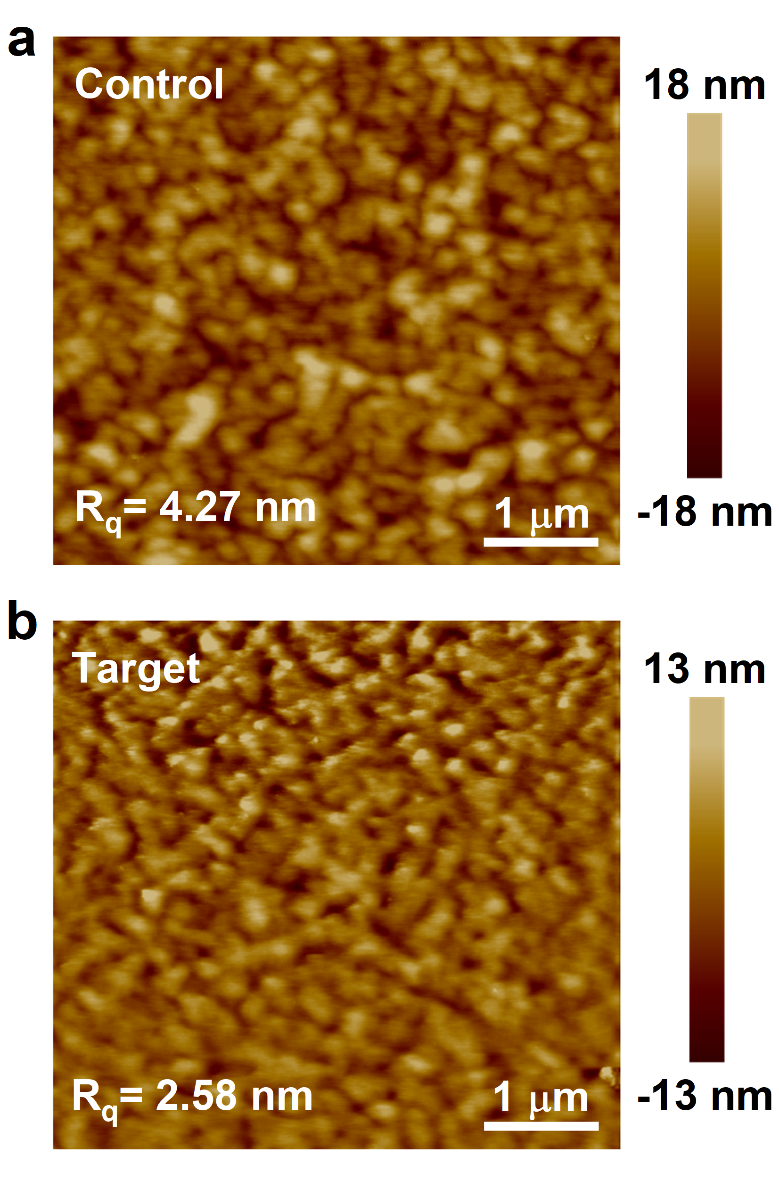


**Fig. S17** AFM topography images of (a) control and (b) target CsPbBr_3_ NPL films.


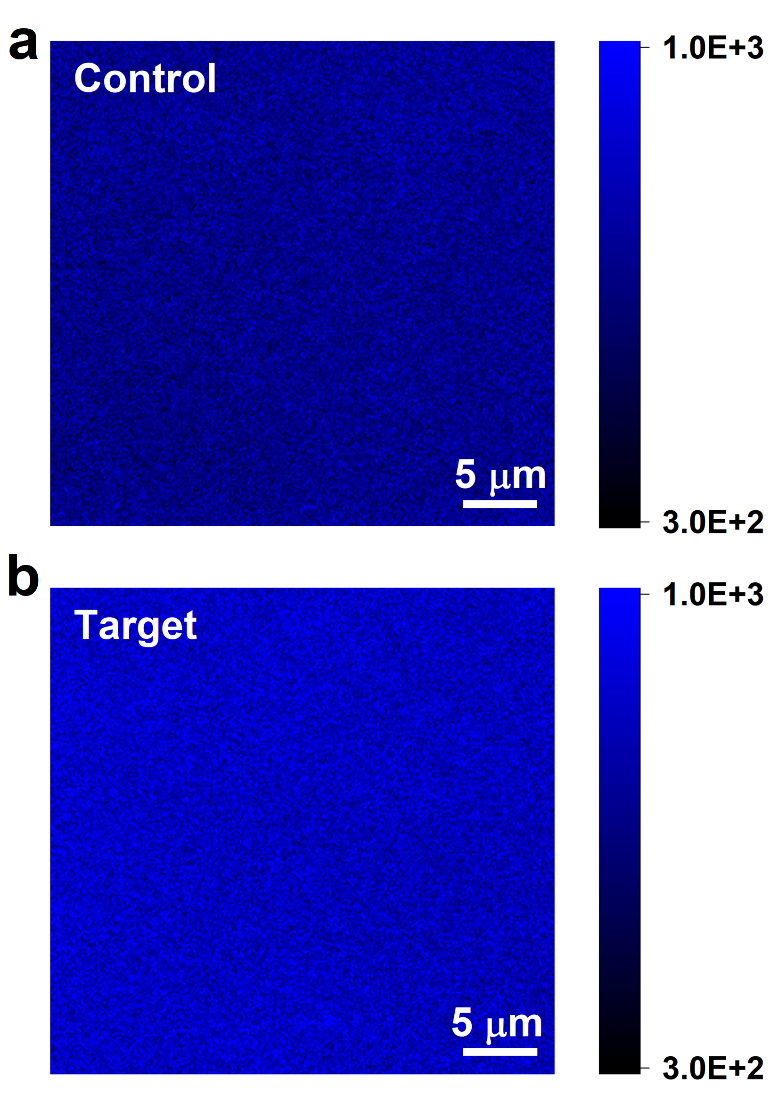


**Fig. S18** PL mapping images of (a) control and (b) target CsPbBr_3_ NPL films.


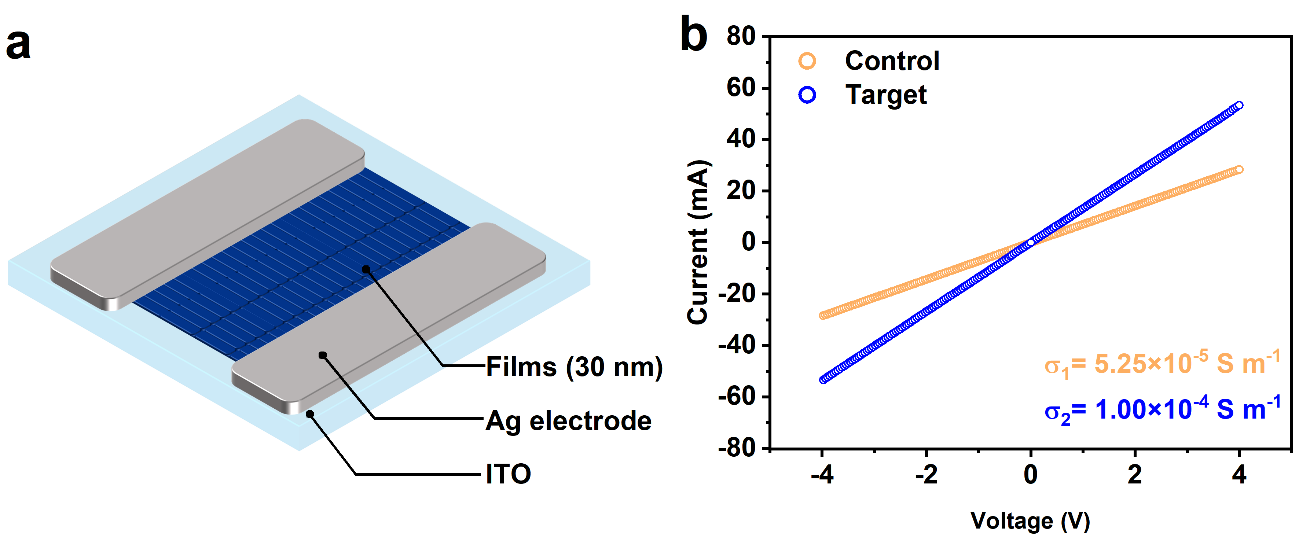


**Fig. S19 (**a) Schematic diagram of the device structure used for conductivity measurements. (b) Current-voltage (I-V) curves of control and target CsPbBr_3_ NPL films.

**Note S7.**

Electrical conductivity was calculated using the following formula:

$$\begin{aligned} \sigma=\frac{IL}{US}\#\left( 11 \right) \end{aligned}$$

where *I* is the current through the film, *L* is the thickness of the film, *U* is the voltage across the film, and *S* is the area of the film.


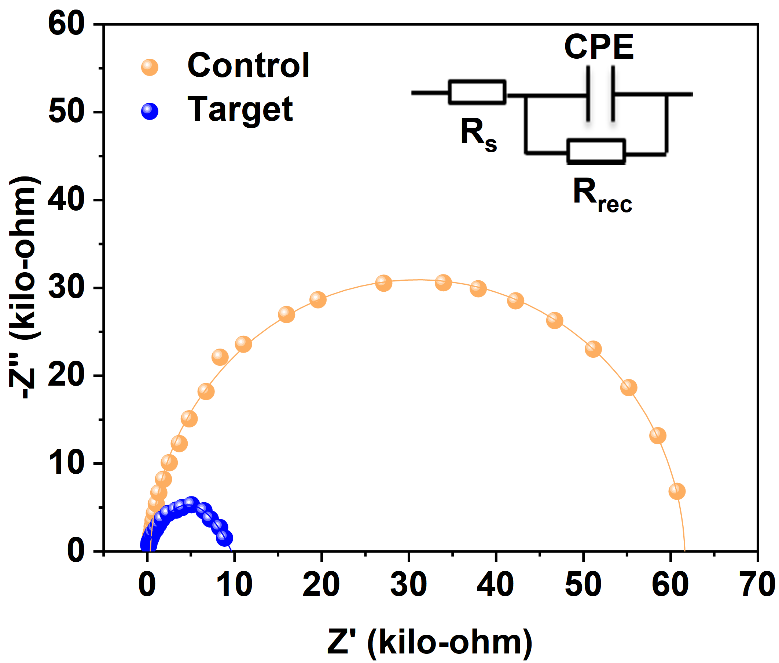


**Fig. S20** Impedance spectra of control and target CsPbBr_3_ NPLs PeLEDs.

As shown in Fig. S20, the impedance spectra were analyzed using an equivalent circuit model comprising a series resistance (R_s_, representing the ITO contact and measurement leads) and a parallel combination of recombination resistance (R_rec_) and a constant phase element (CPE). The fitted Nyquist plots reveal that the recombination resistance R_rec_ decreases markedly from 62 kΩ in the control device to 9.8 kΩ in the target device. This reduction indicates faster carrier recombination kinetics and is consistent with the observed improvements in EL efficiency and reduction of non-radiative recombination loss.

**Reference**

1 Kresse, G. et al. From ultrasoft pseudopotentials to the projector augmented-wave method. *Physical Review B* **59**, 1758-1775 (1999).

2 Perdew, J. P. et al. Generalized gradient approximation for the exchange-correlation hole of a many-electron system. *Physical Review B, Condensed matter* **54**, 16533-16539 (1997).

3 Grimme, S. Semiempirical GGA-type density functional constructed with a long-range dispersion correction. *Journal of Computational Chemistry* **27**, 1787-1799 (2010).

4 Wu, Y. et al. In situ passivation of PbBr_6_^4–^ octahedra toward blue luminescent CsPbBr_3_ nanoplatelets with near 100% absolute quantum yield. *ACS Energy Letters.* **3**, 2030-2037 (2018).

5 Zhang, C. et al. Surface ligand engineering toward brightly luminescent and stable cesium lead halide perovskite nanoplatelets for efficient blue-light-emitting diodes. *The Journal of Physical Chemistry C* **123**, 26161-26169 (2019).

6 Yin, W. et al. Multidentate ligand polyethylenimine enables bright color-saturated blue light-emitting diodes based on CsPbBr_3_ nanoplatelets. *ACS Energy Letters.* **6**, 477-484 (2021).

7 Wang, H. et al. Efficient CsPbBr_3_ nanoplatelet-based blue light-emitting diodes enabled by engineered surface ligands. *ACS Energy Letters.* **7**, 1137-1145 (2022).

8 Shen, W. et al. Efficient pure blue light-emitting diodes based on CsPbBr_3_ quantum-confined nanoplates. *ACS Applied Materials & Interfaces* **14**, 5682-5691 (2022).

9 Liu, H. et al. Efficient and stable blue light emitting diodes based on CsPbBr_3_ nanoplatelets with surface passivation by a multifunctional organic sulfate. *Advanced Energy Materials* **13**, 2201605 (2023).

10 Liu, H. et al. Organic semiconducting ligands passivated CsPbBr_3_ nanoplatelets for blue light-emitting diodes. *ACS Energy Letters.* **8**, 4259-4266 (2023).

11 Huang, Q. et al. Enhancing crystal integrity and structural rigidity of CsPbBr_3_ nanoplatelets to achieve a narrow color-saturated blue emission. *Light: Science & Applications* **13**, 111 (2024).

12 Wang, L. et al. Efficient, color-stable, pure-blue light-emitting diodes based on aromatic ligand-engineered perovskite nanoplatelets. *Nano Letters.* **24**, 7004-7011 (2024).

13 Park, J. et al. Efficient and spectrally stable pure blue light-emitting diodes enabled by phosphonate passivated CsPbBr_3_ nanoplatelets with conjugated polyelectrolyte-based energy transfer layer. *EcoMat* **6**, e12487 (2024).
